# A closed-loop, music-based brain-computer interface for emotion mediation

**DOI:** 10.1371/journal.pone.0213516

**Published:** 2019-03-18

**Authors:** Stefan K. Ehrlich, Kat R. Agres, Cuntai Guan, Gordon Cheng

**Affiliations:** 1 Chair for Cognitive Systems, Department of Electrical and Computer Engineering, Technische Universität München (TUM), Munich, Germany; 2 Institute of High Performance Computing, Social and Cognitive Computing Department, Agency for Science, Technology and Research (A*STAR), Singapore, Singapore; 3 Yong Siew Toh Conservatory of Music, National University of Singapore (NUS), Singapore, Singapore; 4 School of Computer Science and Engineering, Nanyang Technological University (NTU), Singapore, Singapore; University of Pécs Medical School, HUNGARY

## Abstract

Emotions play a critical role in rational and intelligent behavior; a better fundamental knowledge of them is indispensable for understanding higher order brain function. We propose a non-invasive brain-computer interface (BCI) system to feedback a person’s affective state such that a closed-loop interaction between the participant’s brain responses and the musical stimuli is established. We realized this concept technically in a functional prototype of an algorithm that generates continuous and controllable patterns of synthesized affective music in real-time, which is embedded within a BCI architecture. We evaluated our concept in two separate studies. In the first study, we tested the efficacy of our music algorithm by measuring subjective affective responses from 11 participants. In a second pilot study, the algorithm was embedded in a real-time BCI architecture to investigate affective closed-loop interactions in 5 participants. Preliminary results suggested that participants were able to intentionally modulate the musical feedback by self-inducing emotions (e.g., by recalling memories), suggesting that the system was able not only to capture the listener’s current affective state in real-time, but also potentially provide a tool for listeners to *mediate their own emotions* by interacting with music. The proposed concept offers a tool to study emotions *in the loop*, promising to cast a complementary light on emotion-related brain research, particularly in terms of clarifying the interactive, spatio-temporal dynamics underlying affective processing in the brain.

## 1 Introduction

Research on emotion has a longstanding tradition that has garnered interest from a variety of scientific fields, such as psychology, sociology, and neuroscience (e.g., [1–4]). For example, scientists now know that there is at least some degree of independence between emotion processing and attentional mechanisms, that the amygdala is crucial in forming conditioned fear responses, and that evoking certain emotional states biases decision making processes [5]. These findings have shed light on the cognitive, neural, and social factors at play in emotion processing. Despite these advances, existing theories and models of emotion often lack consistency, and are often strongly context-dependent [6]. In addition, the majority of neuroscientific research on affective responses has investigated brain processes involved in mere *recognition* of emotional content, while the brain processes involved in the *induction* and *mediation* of affective states by emotionally-evocative stimuli are not yet well understood, due in part to the difficulty of carefully controlling these types of studies. That is, traditional approaches have elicited important breakthroughs in terms of how we understand affective responses at the behavioral, social, and physiological levels. They have not, however, provided many tools for directly quantifying neurophysiological measures of emotion that can help individuals both detect and modulate their own emotions. This is where the comparatively recent fields of *Affective Computing* [7] and *Brain-Computer Interfaces* (BCI) [8, 9] can offer important insight into the physiology and neuroscience of emotions, as well as cutting-edge techniques. Recently, BCI has been suggested as a “powerful tool for scientific inquiry” of the brain in vivo, most notably for investigating the nervous system’s adaptive capacities during interaction with external stimuli [10]. In particular, BCI has the clear methodological advantage of offering interactive approaches to capturing neural responses that may be used to facilitate self-regulation of affective states in users.

BCI approaches to affective states are of particular importance at this time, not only to advance our understanding of emotion induction mechanisms by investigating the neural signatures of emotions, but also for the myriad possible medical applications [11]. For example, mental diseases, such as mood disorders and depression, have their origin in emotional dysfunctions [12]. Effective techniques to promote emotional regulation are of great importance to the field of medicine; the affective BCI approach has been proposed as one possible route in this direction [13]. Indeed, BCI-based treatment protocols have been proposed for several neurological disorders, such as attention deficit hyperactivity disorder (ADHD) [14] as well as depression in elderly [15].

To aid potential BCI approaches for emotion regulation, *music* is a useful medium, as it is widely acknowledged to be highly effective in eliciting affective responses. Indeed, one of the main reasons people reportedly listen to music is to change or enhance their mood [16]. Therefore, incorporating musical feedback into BCI systems offers great potential for emotion-regulation systems.

Music and emotion: Music is known to express and evoke strong emotions [17–19], and perceiving music implicates a wide spectrum of cognitive brain processes, such as short and long term memory, visual and auditory perception and integration, physiological entrainment, motor planning, and coordination [12]. Because music taps into so many different cognitive resources, it can serve as an outstanding tool for better fundamental understanding of the human brain. As Leng and Shaw [20] stated decades ago: “Since processing of music is a complex set of perceptive and cognitive operations with links to memory and emotions, it offers a window to the understanding of higher brain functions”.

Music is also a culturally robust phenomenon; evidence of musical behavior has been found in every society [21, 22]. Furthermore, music perception starts very early in human ontogeny, with infants demonstrating, for example, rhythmic and melodic discrimination [23]. Scientists have even suggested that music was even a precursor to language development over the course of human evolution [24]. Studies have also found that a basic understanding of *emotional content* in music begins in early childhood [17]. Because music is so widely appreciated, and is often used (informally) by individuals to mediate or enhance their own emotional states, it has great potential to be an effective tool for investigating affective regulation in listeners.

Several prior studies have employed music to trigger specific patterns of brain activity, showing that it can be used as a diagnostic tool for neurological dysfunctions [25] and for treatment of such diseases [26], [27]. Music has also been used to treat depression and anxiety in the elderly, individuals suffering from mental health disorders, and numerous other clinical conditions and diseases (see for example [15, 28–31]). Scientists acknowledge that “A better understanding of music evoked emotions and their neural correlates can lead to a more systematic and effective use of music therapy because dysfunctions in these structures are related to emotional disorder” [12].

Another important feature of music that is particularly advantageous for the BCI context of the present work: Unlike many other types of emotional stimuli that are difficult to manipulate in specific ways over time, such as pictures and videos, music can algorithmically be synthesized, and thus be used to form custom-made emotional stimuli. It can serve as a continuous affective display—a real-time representation of a person’s affective state or desired affective state. The idea to translate human brain activity into sound or music was first proposed about five decades ago, however driven by artistic, rather than scientific objectives. In a public demonstration in 1965 entitled “Music for Solo Performer”, Alvin Lucier translated EEG measures into actuations for simultaneously playing several acoustic percussion instruments [32]. A more contemporary application along these lines has been presented by Hinterberger and Baier [33], who reported an EEG-based device for the sonification of brain-oscillations. Similarly, Miranda et al. presented a concept in 2005 that maps alpha- and beta-bandpower (representing relaxation- and attention-level) onto music control parameters to switch between two different styles of music [34]. In 2011, Makeig et al. [35] conducted a study in which the participant was asked to associate several two-tone musical drone sounds with corresponding self-induced emotions. Related EEG-patterns were captured during a calibration session and subsequently modeled. In a live performance together with a small chamber orchestra, the user was then able to trigger the drone sounds by re-experiencing the initially associated self-induced emotions. A more recent work by Deuel et al. in 2017 [36] utilized EEG alpha- and mu-band oscillations to allow the user to generate different notes of a musical scale based on the level of bandpower activation in real-time. The objectives of the above mentioned works were mainly for artistic purposes, and except for the work by Makeig et al. [35], were not particularly focused on emotional brain processes. Nevertheless, they demonstrate how well music lends itself to being generated in real time based on the user’s emotions, and more specifically, how brain responses may be used to drive music generation.

**Fig 1:**
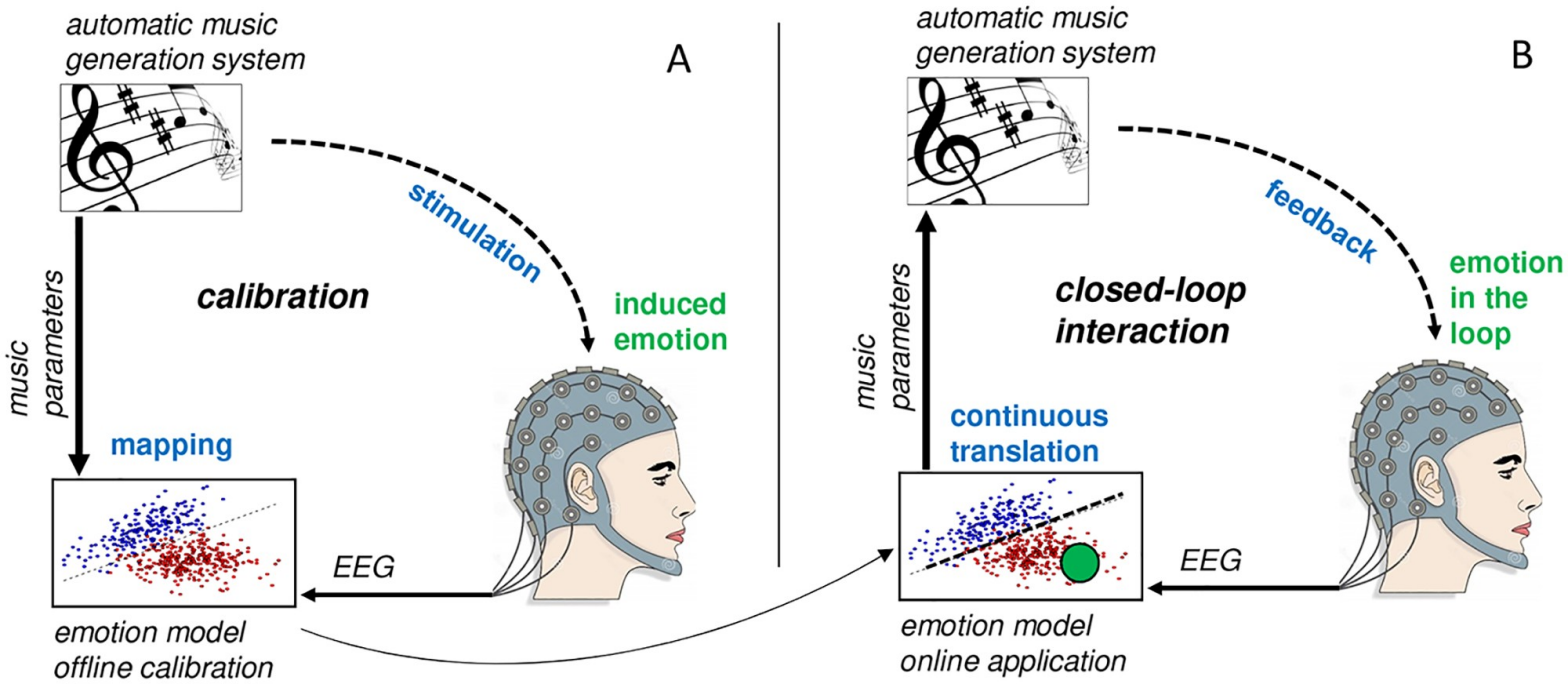
Conceptual illustration of the affective music BCI. (A) During calibration, the user is exposed to automatically generated patterns of affective music, and brain activity is measured simultaneously via EEG. EEG patterns are extracted and used to build a user-specific emotion model. (B) During online application, the obtained model is used to continuously translate the user’s brain activity into affective music, thereby closing the loop through continuous affective brain interactions.

This work presents a novel Brain-Computer Interface (BCI) to feedback a user’s emotional state by means of algorithmically synthesized music in such a way that continuous closed-loop affective interaction can be established. The concept presented here builds upon preliminary work of the authors [37]. The core component of our approach—and the major contrast to previous works—is the utilization of a parameterizable music synthesis algorithm (Fig 1). This algorithm allows for generating musical sequences with seamless and continuous transitions between patterns of different emotional expressiveness. This algorithm is utilized to both ‘calibrate’ the system for EEG-based affect modeling (Fig 1A), and for online application (continuous EEG-based affect translation, as shown in Fig 1B). This approach allows for a seamless transition between the calibration phase and the online application phase as well as high flexibility for developing novel calibration and stimulation protocols.

The remainder of this paper is structured as follows: The next section introduces our proposed affective BCI concept, as well as a description of the technical realization of our concept. The evaluation of the system is described in two sections: Section 2.3 describes a listening study that evaluates the algorithm’s ability to generate synthesized affective music. In Section 2.4, this algorithm is integrated into the proposed affective BCI, and a real-time BCI study for evaluating *affective closed-loop interaction* utilizing our proposed affective BCI. Section 3 provides results of these two studies, which is followed by a discussion and concluding remarks in Section 4.

## 2 Materials and methods

### 2.1 Automatic music generation system

**Fig 2:**
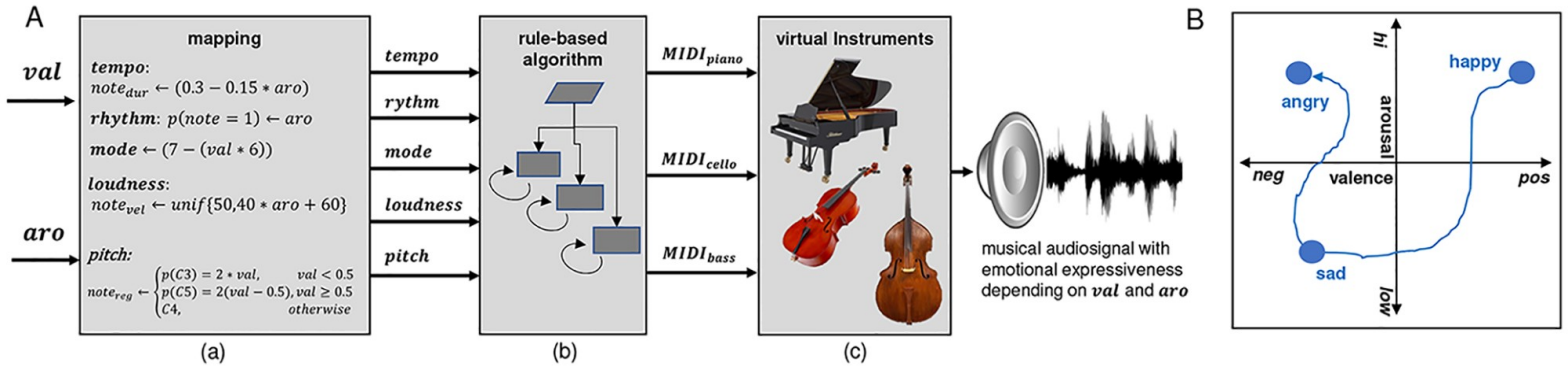
Schematic illustration of the automatic music generation system. (A) mapping of valence and arousal onto music structural parameters (a), state-machine to generate MIDI patterns (b), virtual instruments to translate MIDI patterns into sound (c). (B) Exemplary synthesized musical trajectory through affective space (valence-arousal-model according to [38]).

Music can be thought of as a combination of harmonic, rhythmic and timbre components that change over time. The automatic music generation system was implemented as a rule-based probabilistic algorithm (see Fig 2A(b)), inspired by the work of Wallis et al. in 2008 [39] and 2011 [40], however with widely different technical realization. Our algorithm generates streams of MIDI-events, whereupon the occurrence and type of events are modulated by several continuous control parameters. These control parameters were implemented such that they modulate the musical pattern according to five music structural components, namely: harmonic *mode*, *tempo*, rhythmic *roughness*, overall *pitch*, and relative *loudness* of subsequent notes. According to their settings, different musical patterns are generated. However, these parameters do not inherently allow the generation of emotion-specific musical patterns. The crucial aspect of the technical realization is illustrated in Fig 2A(a): Several psychological studies [41–43] have shown functional relationships between music structural parameters and emotional responses expressed by valence and arousal (see [38]). We employed a subset of functional relationships proposed by Gomez and Danuser in 2007 [43] to map two emotion-related parameters (valence and arousal, val,aro∈[0,1]⊂R) onto the five implemented music structural parameters (input to the algorithm). The music generation system’s input controls and the generated musical patterns are consequently emotion-related. The MIDI-patterns are sent over a virtual MIDI path (MIDI Yoke) to software (ProTools 8 LE) hosting virtual instruments (Fig 2A(c)) that are then translated into sound (playback engine was an AVID MBox3 Mini external soundcard). In total 3 virtual instruments (piano, cello, bass) were controlled with the MIDI-signals, resulting in a musical style comparable with a small classical chamber orchestra (sound examples are available online here: http://ics.ei.tum.de/~ehrlich/affectiveBCI/). The music structural parameters were implemented as described below. The smallest note entity was set to an eighth note whose duration *note*_*dur*_ is determined by the parameter tempo. Tempo was functionally related to arousal according to Eq 1. The number of notes played within one bar was randomly set based on a probability determined relative to the arousal input parameter according to Eq 2. This parameter was called *rhythmic roughness* as it controls the amount of notes being played, with more notes resulting in more complex rhythmicity. The velocity (loudness) of each note was uniform randomly set within a loudness range, whereupon the range is determined by the parameter relative *loudness* of subsequent tones. Relative loudness was functionally related to arousal according to Eq 3. The pitch register wherein one note *note*_*reg*_ is played was also randomly set, such that the probability for high (C5), middle (C4) or low registers (C3) is determined by the parameter *pitch*. Pitch was functionally related to valence according to Eq 4. The parameter **harmonic mode** determines which chords are played within one chord progression. One chord progression consists of four subsequent bars; one chord is played per bar. The tonal key was fixed to C-major, as well as the harmonic progression to I-IV-V-I (among the most common chord progressions in music composition [44]). According to the principle of *Kirchentonleitern*, seven harmonic modes are possible. An order of these modes from positive to negative valence has been proposed by Schmuckler in 1989 [45], namely: 1. Lydian (4th mode), 2. Ionian (1st mode), 3. Mixolydian (5th mode), 4. Dorian (2nd mode), 5. Aeolian (6th mode), 6. Phrygian (3rd mode), and 7. Locrian (7th mode). The resulting chord progression is determined by the selected mode (Lydian mode, e.g. would result in the following chord progression: ||:*F*_*maj*_|*C*_*maj*_|*B*_*dim*_|*F*_*maj*_:||). Harmonic mode follows a 7-step discrete order, and is related to valence in a discrete manner according to Eq 5. Note that all parameters, with the exception of harmonic mode, are continuous.

$$\begin{array}{c} \text{tempo}:\;note_{dur}=0.3-\text{aro}*0.15\subset R \end{array}$$ (1)

$$\begin{array}{c} \text{rhythm}:\;p(note=1)=\text{aro} \end{array}$$ (2)

$$\begin{array}{c} \text{loudness}:\;note_{vel}=unif\left\{50,40*\text{aro}+60\right\}\subset N \end{array}$$ (3)

$$\begin{array}{c} \text{pitch}:\;note_{reg}=\{\begin{array}{cc} p(C3)=2*\text{val} & \;\text{if}\;\text{val}<0.5\\ p(C5)=2*(\text{val}-0.5) & \;\text{if}\;\text{val}\geq 0.5\\ C4 & \;\text{otherwise} \end{array} \end{array}$$ (4)

$$\begin{array}{c} \text{mode}=7-(6*\text{val})\in 1,...,7\subset N \end{array}$$ (5)

The way the music generation system was implemented allows for generating continuous streams of music patterns with particular qualities of emotional expressiveness (parameterizable with the respective input parameters valence and arousal). By gradually changing the input parameters, the algorithm’s generated music patterns flow seamlessly and gradually change in terms of emotional expressiveness. Any desired ‘trajectory’ through the valence-arousal-space is therefore possible, e.g. going continuously from happy over sad towards angry musical expressiveness (see exemplary trajectory in Fig 2B). This principle allows the integration of the algorithm into an online BCI architecture according to our initially proposed concept (Fig 1). The applicability of the automatic music generation system is, however, not restricted to the use case described in this work. The system was also employed in one of our previous works on continuous augmentation of speech with affective synthesized music based on real-time speech emotion recognition [46].

### 2.2 Affective BCI architecture

We developed a functional prototype of our concept by introducing the automatic music generation system into an online BCI architecture. In the following sections we first describe the system calibration, then the procedure of building emotion classification models based on EEG data recorded during calibration, and, finally, how we apply the emotion classification model online to continuously translate the user’s affect into a musical representation using the automatic music generation system.

#### 2.2.1 Calibration

For a first proof-of-concept, we purposely simplified a few aspects of the prototype development. For instance, during the calibration phase, the listener was exposed to three classes of musical excerpts generated by the automatic music generation system, namely: *sad* (low-arousal-negative-valence), *neutral* (intermediate-arousal-intermediate-valence), and *happy* (high-arousal-positive-valence). Out of these three classes of musical excerpts, only two classes (sad, happy) were used for the subsequent modeling step. This simplification allowed us to treat the modeling step as a binary classification problem. The neutral excerpts were introduced as a mediator between the two classes of interest (e.g., to reduce the contrast between subsequently presented excerpts). Each excerpt was presented two times for 20 sec each in a pseudo-randomized order, such that subsequent excerpts were always of different classes. The excerpts were separated by idle periods of 10 sec in which no music was presented. The total calibration time was around three minutes. Brain activity during calibration was measured via a 14-channel Emotiv EPOC EEG system with a sampling rate of 128 Hz.

#### 2.2.2 Modeling

**Fig 3:**
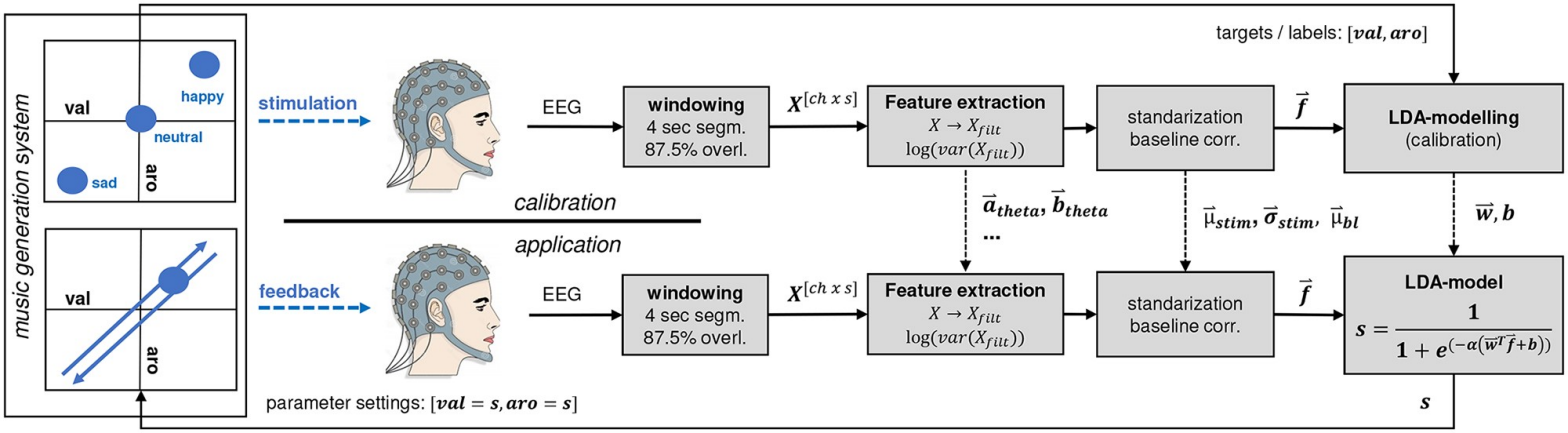
BCI architecture. Signal processing and information flow during calibration (top row) and online application (bottom row) of the system. Note, the dual use of the automatic music generation system in both cases for providing either open-loop stimulation (calibration) or closed-loop feedback (application). The black dashed lines indicate model-specific parameters (filter parameters, standardization and baseline parameters, and classifier parameters) that were obtained during calibration, and utilized during online application.

After presentation of the calibration excerpts, the recorded EEG data were automatically modeled offline in 10 steps (Fig 3, top): (1) The respective listening periods of the two classes of interest and the intermittent idle periods were extracted from the continuous EEG data. (2) The data were further partitioned into segments with a window size of 4 sec and an overlap of 87.5%. In previous in-house pilot experiments, we varied the window size (1, 2, 4, and 8 sec) and empirically found the best system performance for 4 sec. segments. We defined 87.5% overlap as this resulted in a reasonable update rate of 0.5 sec. during online application. (3) The EEG data were filtered into 5 frequency bands by means of 2^nd^ order zero-phase Chebyshev IIR-bandpass filters. Please note that no continuous filtering was performed; zero-phase filtering was conducted on each segment independently after windowing. This approach was chosen to avoid unnecessary delays introduced by filtering. The selection of frequency bands was based on the standardized recommendations [47], namely theta (4-7 Hz), alpha (8-13 Hz), low beta (14-21 Hz), high beta (22-29 Hz) and gamma (30-47 Hz). We excluded delta-band (0-4 Hz) as low frequency components in the EEG are prone to contamination by eye-movement artifacts and slow signal drifts. Furthermore, we separated the beta-band into a low and high components because of its large bandwidth in comparison to the other frequency bands. (4) Spectral band power features *f* were extracted by computing the log-variance *f* = *log*(*var*(*x*_*filt*_(*t*))) of each segment *x*_*filt*_(*t*) for all 14 channels and 5 frequency bands, resulting in a total of 70 features, 80 observations per class, and 120 observations for the idle condition. (7) The observations from the idle condition were averaged, forming a baseline-vector. (8) The baseline-vector was then subtracted from each observation of the feature vector, resulting in class-wise observations with cleared baseline. (9) The baseline-cleared observations from the two classes of interest were then standardized. (10) Finally, the observations were used to build a classification model based on Linear Discriminant Analysis (LDA). The LDA discriminant function is the hyperplane discriminating the feature space corresponding to two classes: $y\left(x\right)={\vec{w}}^{T}\vec{f}+b$, with $\vec{f}$ being the feature vector, $\vec{w}$ being the normal vector to the hyperplane (or weight vector), *b* the corresponding bias. The weight vector and bias were computed by $\vec{w}=\left(\vec{\mu_{2}}-\vec{\mu_{1}}\right){\left(\Sigma_{1}+\Sigma_{2}\right)}^{-1}$ and $b=-{\vec{w}}^{T}\left(\vec{\mu_{1}}+\vec{\mu_{2}}\right)$, with $\vec{\mu_{j}}$ being the class-wise means, and Σ_*j*_ being the class-wise covariance matrices.

#### 2.2.3 Online application

During online application, the previously built LDA-model was used as a translator of EEG signals into input parameter settings for the automatic music generation system (Fig 3, bottom). Incoming EEG data (in segments of 4 sec with 87.5% overlap) were used for online feature extraction and classification. Identical to the calibration phase, 70 band-power features were extracted from one segment of EEG data (see processing pipeline in section 2.2.2). The baseline vector and the standardization parameters obtained during the offline modeling phase were used to clear the baseline and subsequently rescale the feature vector. According to Eq 6, the feature-vector $\vec{f}$ was then applied to the formerly built LDA-model to calculate the distance from the decision hyperplane separating the two classes of interest, given by the normal vector $\vec{w}$ and bias *b* of the hyperplane. The sigmoid function scales the LDA-classifier output resulting in the final control signal *s* ∈ (0, 1) (referred to as ‘score’ in the remainder of this paper). This controls the system reactivity; in other words, the steepness, determined by sigmoid function parameter *α*, controls how sensitive the score changes with regard to changes in the feature vector. This parameter was set to *α* = 2, which was determined empirically based on subjective assessment of the system reactivity (perceived feedback latency) in previous pilot experiments. Each score update (one per 0.5 sec) was subsequently translated into an update of the music generation system’s input parameters, with *val* = *s* and *aro* = *s*. Closing the loop was achieved by playing back the resulting musical patterns to the subject. This enabled the subject to interact with the affective musical feedback along a continuous scale between the affective states *sad* (low-arousal-negative-valence) and *happy* (high-arousal-positive-valence).

$$\begin{array}{c} s=\frac{1}{1+e^{\left(-\alpha \left({\vec{w}}^{T}\vec{f}-b\right)\right)}},\;s\in \left(0,1\right) \end{array}$$ (6)

### 2.3 Listening study: Evaluating the affective responses of the algorithmic compositions

The purpose of this study was to investigate whether participants’ affective responses to the generated musical patterns were in accordance with the corresponding valence and arousal settings of the music generation system. That is, the listening study was meant to evaluate and validate the functionality of the automatic music generation system, a prerequisite for justifying its use in the online BCI system.

#### 2.3.1 Listening study participants

Eleven healthy participants (age: 26.9±3.4 yrs; 7 males, 4 females) participated in this study. The group consisted of 4 musicians (with at least 5 years of formal musical education) and 7 non-musicians. All participants reported regularly listening to music at least several days per week. Music preferences ranged across classical, pop, rock genres, hip-hop, latin, electronic, and experimental music. Regardless of musical expertise or listening preferences, all participants were given the same instructions prior to the experiment. In addition, all participants signed a data collection consent form prior to the study. The study was approved by the Institutional Review Board (IRB) of the National University of Singapore (NUS) under reference number 08-036.

#### 2.3.2 Listening study stimuli

The automatic music generation system’s input parameters span a two-dimensional emotional scale (valence-arousal model). In total, 13 uniformly-distributed locations in the valence-arousal space were selected to be presented as stimuli. The individual stimuli for each of the 13 locations were specific musical patterns arising from the settings of the music generation system’s input parameters: {val,aro} = [{0,0}; {0,0.5}; {0,1}; {0.25;0.25}; {0.25,0.75}; {0.5,0}; {0.5,0.5}; {0.5,1}; {0.75,0.25}; {0.75,0.75}; {1,0}; {1,0.5}; {1,1}]. The order of presentation of the stimuli was pseudo-randomized for each individual participant.

#### 2.3.3 Listening study experiment protocol

The experiment took place in a quiet room without visual or auditory distraction. Music was played back via an AVID Mbox Mini (external sound-card) and Sennheiser earphones. The volume was set to a comfortable listening volume and participants were allowed to manually correct the volume at the beginning of the experiment to their preferred level. The participants were first instructed (verbally and by means of an instruction text) about the experiment setup, and then completed a brief questionnaire capturing demographic information and music preferences. Next, the 13 music excerpts were consecutively presented to the participants for 30 sec each. Participants were asked to keep their eyes closed during music-listening in order to avoid distraction. After each excerpt, the participants were asked to rate their emotional responses by means of the Self-Assessment Manikin (SAM) [48], which is essentially a visual representation of a 9-point Likert scale. Participants were allowed to take as long they desired to make each rating. The experiment was approximately 15 minutes in duration per participant.

### 2.4 BCI study: Evaluating the affective closed-loop interaction

#### 2.4.1 BCI study objective and hypothesis

The purpose of this pilot study was to investigate to what extent participants were able to gain control over the music feedback, i.e., voluntarily change the music feedback by modulating their brain activity accordingly. We were interested in what strategies participants would develop to attain such voluntary modulations of the music feedback. Gaining control over the musical feedback would imply that participants are able to effectively mediate their own emotional states; this finding would support the usability of BCI system to foster self-regulation of affective responses.

#### 2.4.2 BCI study participants

Five healthy participants (age: 27.8±5.0, all males, all right-handed) participated in this study. Four participants had no formal education in music. The remaining participant had six years of formal musical training, but had not played music in the seven years prior to this experiment. All participants reported regularly listening to music more than a few minutes per day. Music preferences varied between rock, pop, and more specific music genres such as electronic music, folk, and hip-hop. All participants took part voluntarily and gave written consent. The study was approved by the Institutional Review Board (IRB) of the National University of Singapore (NUS) under reference number 08-036.

#### 2.4.3 BCI study experiment protocol

**Fig 4:**
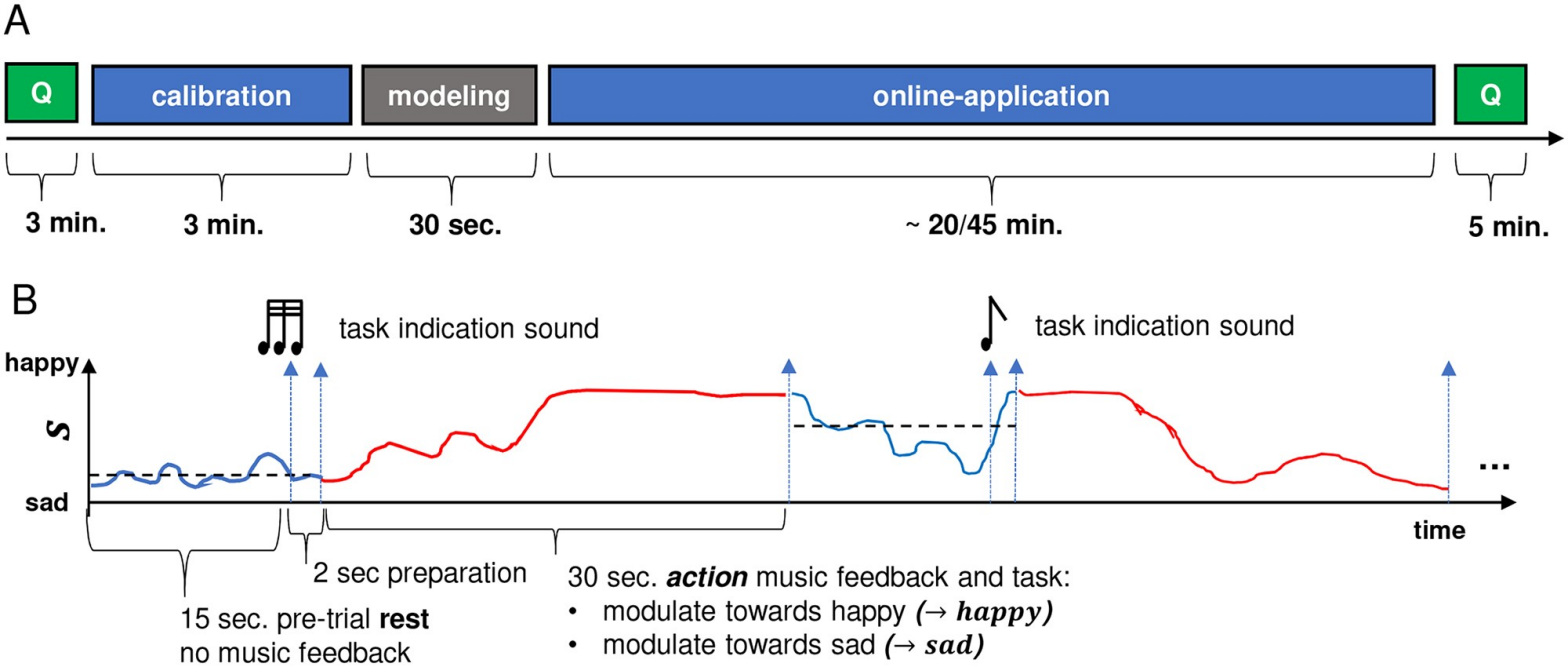
BCI study experiment protocol (A) and trial structure (B). (A) Each session began with a questionnaire, followed by a calibration phase and subsequent modeling of the emotion classifier. Afterwards, the main part of the experiment, the online application phase, was conducted. The experiment ended with a second questionnaire. (B) A single trial started with a resting period of 15 sec in which no music feedback was presented to the participant. The participant‘s EEG was recorded in the background, and the average score was used to assign, on every trial, one of the two tasks (modulate towards happy or sad) to the participant. The subject then performed the task for a duration of 30 sec (action period).

The experiment took place in a quiet room without visual or auditory distractions. EEG data was recorded with a 14-channel emotiv EPOC EEG system. While signal quality and spatial resolution of the emotiv EPOC is lower than that of high-density research-grade gel-based EEG system, a number of publications have demonstrated that the emotiv EPOC system can successfully be employed in different experimental paradigms [49, 50], including neurofeedback applications [15]. In contrast to research-grade EEG systems, the emotiv EPOC system is inexpensive, requires only brief preparation time (around 5 min), is easy to handle (no professional training is needed), supports wireless data transmission (high mobility), and has a comparably unobtrusive design. This offers clear methodological advantages when deploying and validating our approach outside of the laboratory in the future (e.g., in elderly care centers). EEG electrodes were arranged according to the international 10-20 system [51] (AF3, AF4, F7, F8, F3, F4, FC5, FC6, T7, T8, P7, P8, O1, O2) and all leads were referenced to the average of two reference channels located at the locations P3 and P4. The sampling rate was fixed to 128 Hz. Signal quality was checked with the emotiv TestBench software prior to the start of the experiment. Sensors were adjusted until connectivity reached the ‘green’ level (corresponding to an impedance of < 220*k*Ω according to [50]). The participants were seated on a comfortable chair, and music was played back via an AVID Mbox Mini (external sound device) and Panasonic earphones. The music volume was set to a comfortable listening volume, and participants were allowed to manually adjust the volume of the music at the beginning of the experiment to their preferred level. The experiment protocol is depicted in Fig 4A. The participants were instructed (verbally and by means of written instructions) about the experiment procedure, and then completed a questionnaire capturing demographic information and music preferences. Furthermore, participants were asked to perform a self-assessment of their current mood based on a custom-made questionnaire with 14 opposing emotional attributes on a 7-point scale (e.g. *stressed* versus *relaxed*). The experiment started with a 3-minute calibration phase, followed by automated building of the participant individual emotion classification model based on the recorded EEG data during the calibration phase (c.f. Section 2.2.2 for details about technical implementation). The participants were deliberately not given any hints on how to achieve the feedback modulations. Instead, the participants were asked to develop an individual strategy, that they were allowed to change throughout the experiment. According to the continuous metric between high-valence-high-arousal (happy) and low-valence-low-arousal (sad), participants were asked to perform two different tasks: *modulate music feedback towards happy patterns* and *modulate music feedback towards sad patterns*.

A detailed description of the experimental design is shown in Fig 4B. One trial consisted of a resting-period of 15 sec and a subsequent action-period of 30 sec. Although participants were not explicitly instructed to try to modulate the musical feedback through the use of emotion imagery, we hypothesized that they would employ this type of approach. Therefore, we based the durations of the resting and action periods on a study using emotion imagery by Sitaram et. al in 2011 [52]. They specified 24 seconds for the emotion imagery task and 4.5 seconds for the resting period. During the resting period, no music feedback was provided; the participants had no specific task and were asked to relax and prepare for the next trial. The score was acquired during this resting period, and the data were then averaged to determine which of the two tasks the participant would be given during the action-period. When the average resting-score tended towards a sad state (< 0.5), the task *modulate towards happy* was given. Conversely, when the average resting-score tended towards a happy state (>= 0.5), the task *modulate towards sad* was given. Participants were informed about which of the two tasks they had to perform two seconds before the start of the action period. The indication of the task was realized by means of two different emotionally neutral indication sounds with the same duration, pitch, and loudness-level: three subsequent short tones indicated the task ‘modulate towards happy‘, and one long tone indicated the task ‘modulate towards sad‘. The action period started with the onset of music feedback and lasted for 30 seconds, during which participants tried to modulate the music feedback towards the desired state according to the given task. The end of the action period marked the start of the subsequent resting period of the next trial. Participants were asked to sit still and limit their movement, keeping their eyes closed throughout the entire online application phase (including the resting and action periods).

Each participant took part in the BCI experiment twice (on two separate days). In the first session, the participant performed 20 interaction trials (approximate duration of 15 min) to become comfortable with the environment and the experimental setup. In the second session, participants were asked to perform 50 interaction trials (approximate duration of 38 min). In each session, and for each participant, the emotion classification model was (re-)calibrated. The preparation time (questionnaires and calibration) was around 10 min and identical in both sessions; thus the total duration of session 1 was around 25 min (15 min online interaction) and of session 2 around 50 min (38 min online interaction). At the end of each session, participants were asked to describe their experience and strategies while performing the music feedback modulations verbally and by means of a short written text.

## 3 Results

### 3.1 Listening study

**Fig 5:**
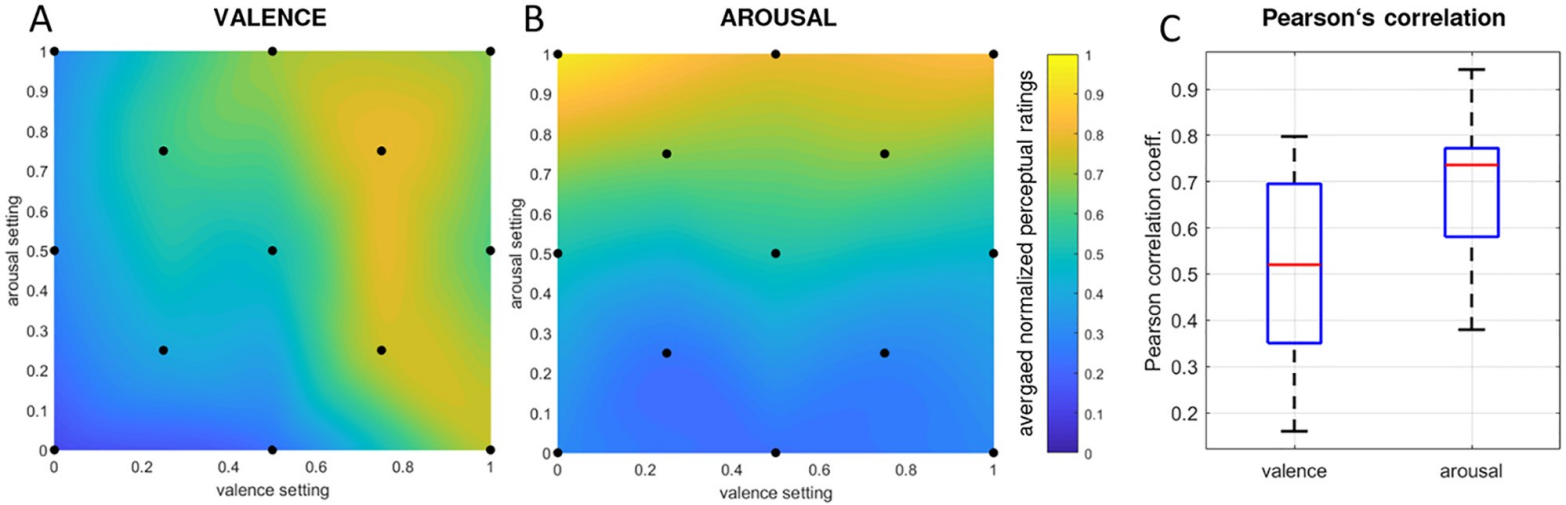
Listening study perceptual ratings. (A) Interpolated valence- and (B) arousal-ratings averaged across subjects. (C) Distribution of per-subject Pearson correlation coefficients between perceptual ratings and the music generation system‘s parameter settings for valence and for arousal.

We discuss below the results of the listening study, where participants provided arousal and valence ratings for each musical excerpt (c.f. Table 1). The raw data of perceptual ratings revealed varying means across participants, e.g. some participants had a bias towards lower ratings and others a bias towards higher ratings. These variations might have been a result of the participants using different reference frames to make their ratings, possibly due to varying music preferences. In order to normalize these variations we rescaled the perceptual ratings of each participant between 0 and 1 for valence and arousal separately. Fig 5A shows the averaged interpolated valence ratings across all presented excerpts. The valence ratings seemed to be influenced by arousal such that excerpts with perceived low arousal produced a bias towards negative valence, whereas the opposite effect is visible for high arousal. Although the results are not optimal due to non-independent valence and arousal ratings, the ratings generally reflected an increase of rated valence relative to an increase of the target values for valence.

**Table 1:** Listening study results. The music generation system’s parameter settings (target valence/arousal) and corresponding perceptual ratings for valence and arousal (MEAN±SD, n = 11).

	target		ratings	
No.	*val*	*aro*	*val*	*aro*
1	0	0	0.12±0.12	0.30±0.32
2	0	0.5	0.30±0.19	0.51±0.35
3	0	1	0.29±0.31	0.95±0.15
4	0.25	0.25	0.39±0.34	0.25±0.26
5	0.25	0.75	0.58±0.29	0.67±0.28
6	0.5	0	0.23±0.18	0.23±0.28
7	0.5	0.5	0.50±0.27	0.52±0.28
8	0.5	1	0.69±0.28	0.80±0.21
9	0.75	0.25	0.74±0.32	0.29±0.24
10	0.75	0.75	0.79±0.23	0.68±0.21
11	1	0	0.73±0.26	0.29±0.24
12	1	0.5	0.62±0.33	0.45±0.24
13	1	1	0.67±0.27	0.83±0.20

In contrast to the valence ratings, arousal ratings were nearly independent from perceived valence (Fig 5B). An increase in the target arousal setting is clearly reflected in an increase in the perceived average arousal. Participants’ arousal ratings nicely reflected the music generation system‘s target values for arousal, as hypothesized. The relatively high standard deviations (c.f. Table 1) may be explained by considerably large variations in participants’ cultural background, musical education, and listening preferences, not to mention the relatively small sample size. Furthermore, the understanding and interpretation of the SAM scheme may have differed slightly from participant to participant. Additionally, for each individual participant, we computed Pearson‘s linear correlation coefficients between the perceptual ratings and the corresponding music generation system‘s input parameters of all 13 excerpts, for valence and arousal ratings separately. Fig 5C shows the distribution of the correlation coefficients across participants (n = 11), with a median correlation of *r*_*val*_ = .52 for valence, and of *r*_*aro*_ = .74 for arousal. For valence ratings, significant correlation coefficients (*p* < 0.05) with target valence were reached in 5 out of 11 participants; for arousal ratings, significant correlation coefficients with target arousal were reached in 8 out of 11 participants.

### 3.2 BCI study

#### 3.2.1 Evaluation of offline model performance

In order to obtain an estimate of the performance of the individual models built prior to the start of the online application phase, we performed 10-fold cross-validations (CV) on the calibration data. Because the calibration data contained only two trials per class, we were unable to perform trial-based cross-validation that would yield meaningful results. Therefore, we decided to partition the data into non-overlapping windows of 1 second in duration. This produced 80 observations (40 per class). We performed the cross-validation by splitting the data into 10 folds, where 9 folds were used for training and the remaining fold was used for testing the model. The folds were then shuffled 10 times such that all folds were used once for testing. The entire procedure was repeated 100 times to obtain a stable estimate of the average performance, resulting in 1000 individual test results, which were then averaged for every model. The averaged results for each model (for every participant/session combination) are reported in Table 2. The average accuracy (i.e., correctly classified samples) across all participants is 58.4% for session S01 with an avg. AUC of 0.58 and 64.9% for session S02 with an avg. AUC of 0.65. The binary chance-level threshold given the number of observations per participant (n = 80) and equal prior probabilities is 58.75% (based on the binomial inverse cumulative distribution function with *p* < 0.05). Therefore, the results were significantly higher than chance for three out of five participants (P03, P04, P05) in session S01, and significantly higher than chance for all four out of five participants in session S02 (P01, P03, P04, P05). The improvement in average cross-validation performance from session s01 to s02 is likely related to participants becoming more familiar with the stimuli and the experimental procedure, resulting in more coherent response data. Nevertheless, the results generally support the validity of the acquired data and the modeling approach employed. It is worth noting that offline model performance does not directly translate into the model‘s performance during online-application, because the listener *in the loop* may be *adapting* to the music feedback during interaction.

**Table 2:** Offline model performance results. Offline model performance based on 100-times-10-fold cross-validation for all subjects (P01-P05) and sessions (S01 and S02). Offline decoding performance is expressed in form of percentage of correctly classified observations per class (happy and sad), overall correctly classified instances (ACC) and via the area under receiver operator curve (AUC).

	S01				S02			
	happy	sad	ACC	AUC	happy	sad	ACC	AUC
P01	49.1%	45.6%	47.3%	0.47	72.6%	76.4%	74.5%	0.75
P02	60.6%	50.7%	55.8%	0.56	61.4%	53.9%	57.6%	0.58
P03	72.1%	52.5%	62.3%	0.62	65.7%	68.0%	66.8%	0.67
P04	65.4%	68.6%	67.0%	0.67	64.5%	62.9%	63.7%	0.64
P05	51.9%	67.6%	59.7%	0.60	63.7%	59.9%	61.8%	0.62
AVG	59.8%	57.0%	58.4%	0.58	65.6%	64.2%	64.9%	0.65

#### 3.2.2 Modulation performance results

After the experiment, participants were asked to describe their strategies verbally and in the form of a short written text. All participants reported strategies based on retrieval of emotional mental images or episodes from the past as well as events they look forward to in the future. In addition, for the ‘modulation towards sad’ task, participant P01 reported in the second session to have successfully used the strategy of self-inducing a “meditative state of mind”, rather than retrieving memories.

**Fig 6:**
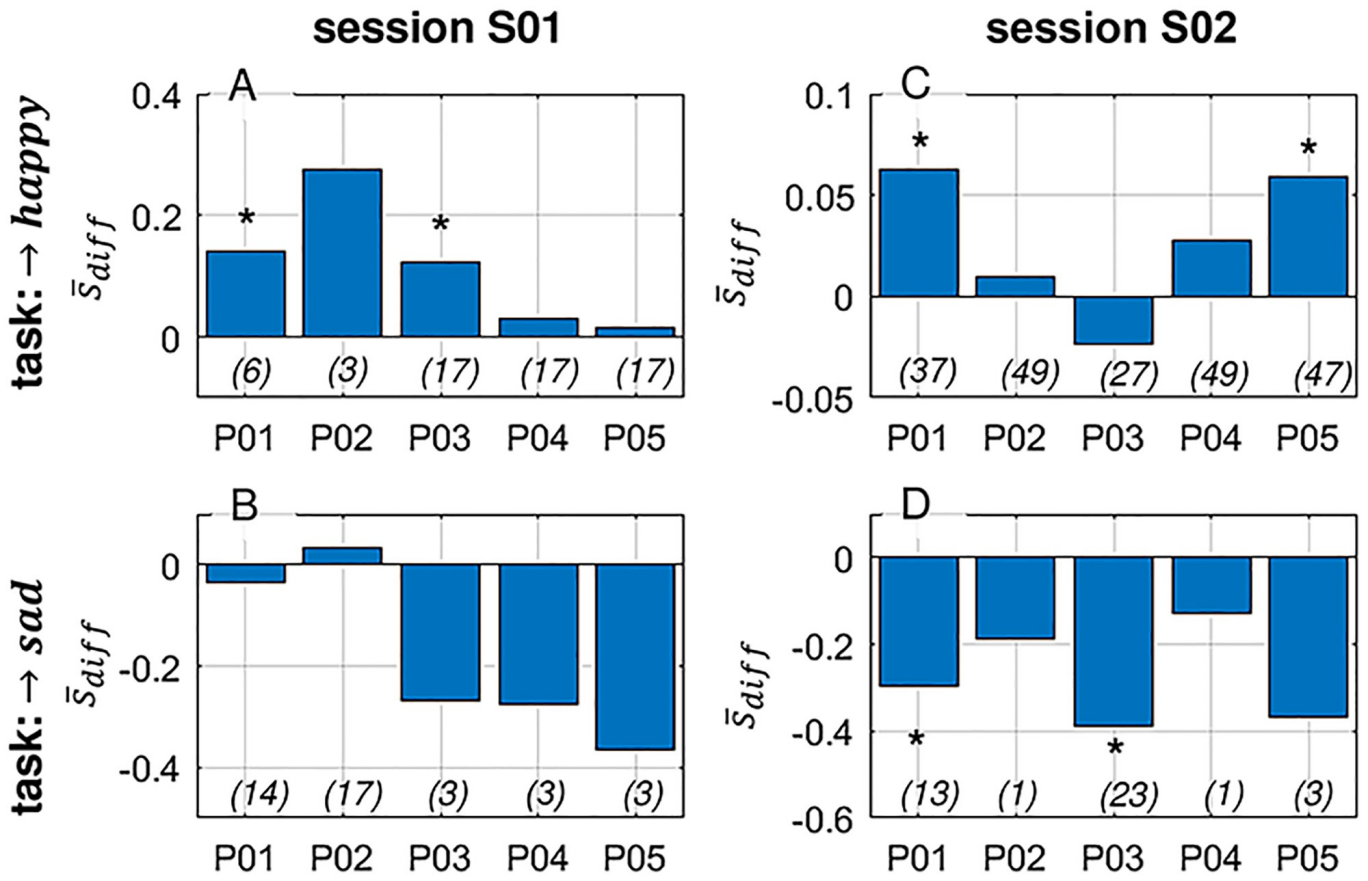
Average modulation performance. The bars represent the mean difference ${\bar{s}}_{diff}$ between the model score of the action period minus the corresponding resting period, averaged across trials (Eq 11) separated in sessions (S01: A and B; S02: C and D) and task (→ *happy*: A and C; → *sad*: B and D). Numbers in parentheses denote the number of trials for the respective participant/task/session combination and the asterisks (*) those combinations with significant modulation performance.

In order to quantitatively assess the participants’ performance in the given tasks, we separated subsequent observations of the model output (score *s*) into resting and action periods for each trial and task separately. The score observations were then averaged into two scalar values per trial: the mean score of the resting-period, and the mean score of the action period. For each individual participant and session, we then performed either a left-tailed (for the ‘→ sad’ trials) or a right-tailed (for the ‘→ happy’ trials) paired t-test, testing the deviation between action and rest across all trials per participant and session according to the given task (see Fig 6). To distinguish between good and poor performance, we used a threshold at the marginal significance level of *p* < 0.1. Surprisingly, although a baseline-correction was performed during the online application phase using the acquired baseline-vector of the calibration phase, most participants developed a bias towards one of the two affective states. This resulted in imbalanced trials in terms of the task distribution for 7 of 10 participant-session-combinations. Therefore, part of the data could not be analyzed using the proposed method due to too few trials per specific task. However, 3 of 5 participants showed significant modulation performance in either one or both tasks. Interestingly, the only musically educated participant (P01) performed best in both sessions and achieved significant modulation performance in both tasks in the second session. Yet, paradoxically, the model built for P01/S01 had very low offline performance compared to other participant-session combinations (see Table 2). The fact that P01 performed relatively well in S01 may indicate that this subject was able to successfully adapt and compensate for the insufficiencies of the model during the online testing phase. Fig 6 shows the average modulation performance in form of the mean difference ${\bar{s}}_{diff}$ between the model score of the action period minus the corresponding resting period, averaged across trials (Eq 11). One can observe that in almost all participant/session/task-combinations, successful modulations were performed, except for P02/S01/→ *sad* and P03/S02/→ *happy*. However, the modulations were only statistically significant in some participant/session/task-combinations (denoted with an asterisk (*)).

#### 3.2.3 Analysis of relationships between modulation performance and mood self-assessment

Besides the objective performance measures obtained in the previous analysis, we were interested in whether observed performance variations among participants and sessions are explained by subjective mood self-assessment ratings provided by the participants before the start of each session, e.g. whether measures of self-assessed mood predicted subsequent performance in the interaction tasks.

For each participant-session-combination, we designed three objective performance measures: *resting mean (RM)*, *action mean (AM)*, and *total deviation (TD)*, as shown in Eqs 7–10, where *s* denotes the score and *n* denotes the total number of task- and period-specific score observations and *m* the number of trials of the corresponding condition.

$$\begin{array}{c} RM=\frac{1}{m}\sum_{i=1}^{m}\frac{1}{n}\sum_{j=1}^{n}s_{rest} \end{array}$$ (7)

$$\begin{array}{c} AM=\frac{1}{m}\sum_{i=1}^{m}\frac{1}{n}\sum_{j=1}^{n}s_{action} \end{array}$$ (8)

$$\begin{array}{c} TD_{happy}=AM_{happy}-RM_{happy} \end{array}$$ (9)

$$\begin{array}{c} TD_{sad}=RM_{sad}-AM_{sad} \end{array}$$ (10)

$$\begin{array}{c} {\bar{s}}_{diff}=\frac{1}{m}\sum_{i=1}^{m}\left(\frac{1}{n}\sum_{j=1}^{n}s_{action}-\frac{1}{n}\sum_{j=1}^{n}s_{rest}\right) \end{array}$$ (11)

We computed Pearson‘s correlation coefficients between the 6 performance measures and the mood self-assessment ratings across all participant-session combinations. The results for mood assessment ratings that significantly correlated with RM, AM, and/or TD are shown in Table 3. The analysis revealed that participants performed larger modulations towards the sad condition if they felt concentrated (rather than distracted) and angry (rather than content) prior to start of the experiment. In addition, a tendency for lower resting mean was discovered when participants felt more calm (rather than excited) before the study. The resting and action means in the → *happy* task correlated with the level of tiredness vs. awakeness reported by the participants prior to the experiment. As a result, for this condition, reports of feeling exhausted, scattered, and tired prior to the study biased the resting and action means to be higher.

**Table 3:** Correlation analysis results. Correlation coefficients, across all participant-session combinations for task ‘modulate towards happy’ (→happy) and task ‘modulate towards sad’ (→sad), between performance measures (resting mean (RM), action mean (AM), total deviation (TD)) and mood assessment ratings (for the six significant mood scales of 14 total ratings made by participants).

	→ happy			→ sad		
	*RM*	*AM*	*TD*	*RM*	*AM*	*TD*
exhausted vs. refreshed	-.59	-.61				
calmed vs. excited				-.44		
distracted vs. concentrated						.67*
scattered vs. focused	-.57	-.56				
angry vs. contented						-.56
tired vs. awake	-.61	-.68*				

Note: Correlations are provided above that have at least marginal significance (*p* < 0.1). Asterisks (*) denote a significant correlation of *p* < 0.05.

#### 3.2.4 Preliminary results indicating brain activity modulation

**Fig 7:**
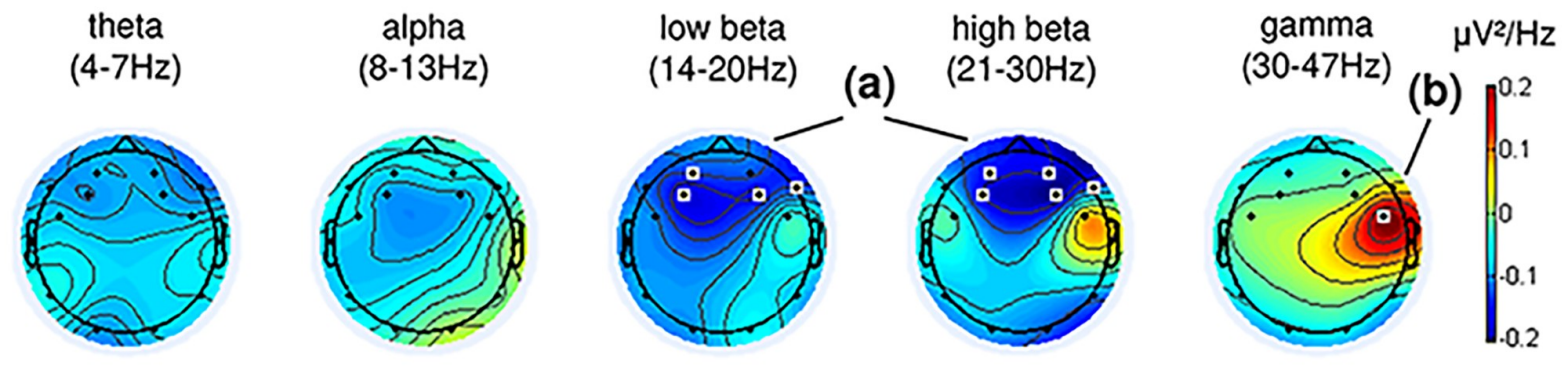
Brain activity modulations during online application in task → happy averaged across participants P01, P04, and P05. Music feedback modulations toward the happy state were accompanied by significant power decrease in beta band over frontal areas (a), as well as an increase in gamma power over the right hemisphere (b). The highlighted channels indicate Bonferroni-corrected statistically significant modulations comparing action and resting periods (action minus rest), (*p* < 0.05).

For our final analysis, we examined participants’ brain activity while performing the modulation tasks. The objective of this analysis was to support the behavioral results reported in the previous section with corresponding electrophysiological results. Specifically, we investigated whether certain kinds of EEG oscillatory brain activity were associated with particular emotion states, as emotion states have been associated with different EEG correlates in the literature (for a review see [53]). In the frequency domain, variations in alpha power have been associated with affective valence [54] and discrete emotions such as happiness, sadness, and fear [55]. Beta band power over right temporal sites has been shown to correlate positively with affective valence [56], and gamma band power modulations have been related to discrete emotions such as happiness and sadness [57–59]. Hence, to support the assumption that participants performed intentional music feedback modulations by self-inducing corresponding emotions, we hypothesize here that feedback modulations were accompanied by changes in emotion-relevant EEG correlates, especially in the alpha, beta, and gamma bands. For this analysis, we focused on session S02, and the task → *happy*, because all participants performed a sufficiently large number of trials (at least 27 times) in this task. Furthermore, only subjects with at least marginally significant modulation performance for the task → *happy* in session S02 were included in this analysis. This criterion excluded participants P02 and P03, leaving participants P01, P04, and P05 for the analysis. We performed a pseudo-online analysis by processing the continuous raw EEG data as in the online application of the original experiment: 4-second long EEG segments were fed into the model segment-by-segment with an overlap of 87.5% in the processing chain (c.f. Section 2.2.3). We then extracted all data segments from the → *happy* (action-period) task, as well as all segments belonging to their corresponding resting periods. For the analysis all data from the respective subjects and task were used; no artifact removal was performed. On each trial, we computed the mean bandpower across all segments of the action-period and subtracted the mean bandpower from all segments of the corresponding resting period. This resulted in one observation per trial (action minus rest) and 70 features in total (14 channels x 5 frequency bands). We then performed within-participant standardization of the observations in order to minimize inter-participant variations. Finally, we performed Bonferroni-corrected multiple t-tests on all features (*p* < (0.05/70)) in order to test for significant differences. The results are depicted in Fig 7 and show that music feedback modulations towards the happy state were accompanied by significant power decrease in beta band over frontal areas, in particular in the high beta band (21-30Hz), as well as a significant increase in gamma band power over the fronto-central right hemisphere. These effects are largely driven by subjects s01 and s05, who exhibited similar topographic plots for all frequency bands. In s04, decreased frontal beta power and a general increase in gamma power are also observable, but are weaker than in s01 and s05.

**Fig 8:**
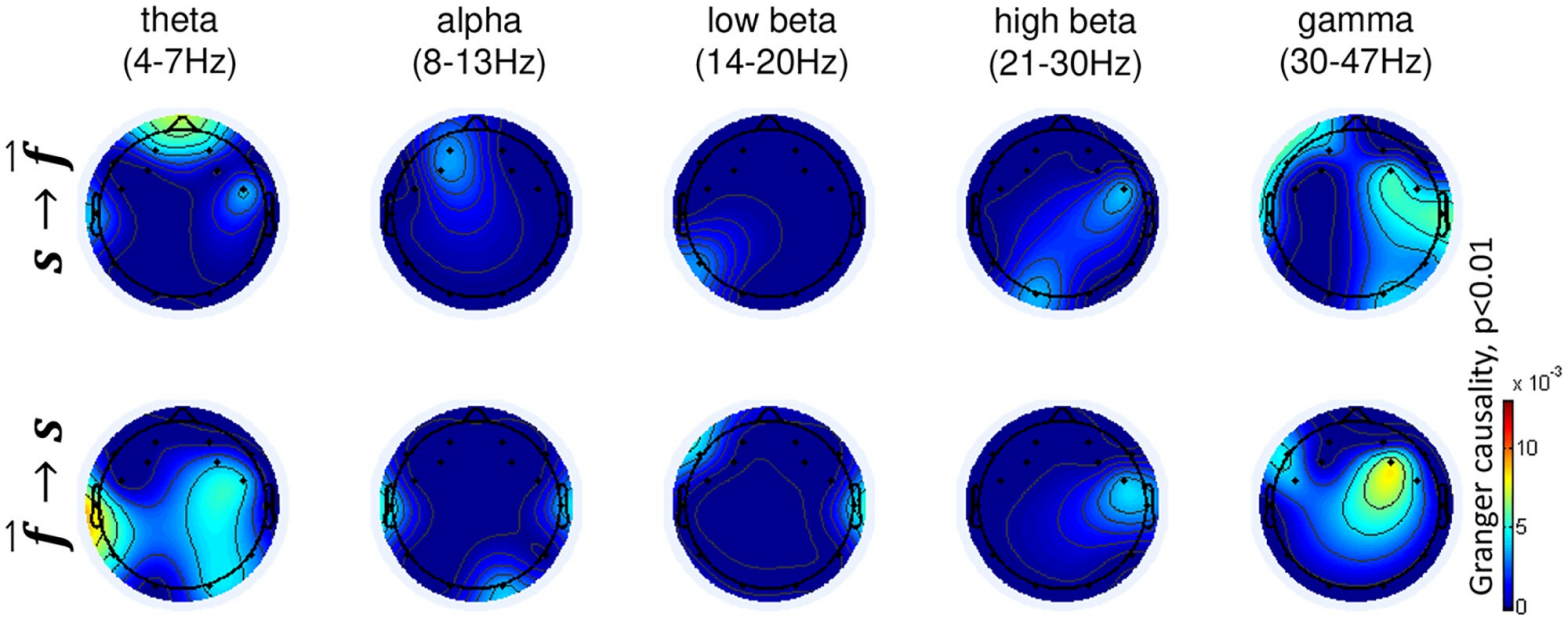
Granger-causal information flows during task →happy in session S02 jointly computed for P01, P04, P05 (participants with good modulation performance). $s\to \vec{f}$ indicates causal flow from feedback to brain activity features (top row) and $\vec{f}\to s$ from brain activity features to feedback (bottom row). The g-causalities are statistically tested for significance (*p* < 0.01) and Bonferroni-corrected.

Granger causality: The closed-loop experimental design allowed us to conduct an exploratory causality analysis to elucidate which neural modulations may have likely contributed to changes in music feedback (and which modulations resulted from hearing the music). One may argue that the significant modulations observed in participants P01, P04, P05 merely reflect a reaction to the onset of the presentation of the music stimuli rather than an effect that reflects a bi-directional interaction between brain activity and musical feedback. To account for this possibility, we performed a causality analysis to investigate whether brain activity modulations caused the feedback to change, whether the feedback triggered a brain response, or whether bidirectional causal relationships were present. For brain activity modulations that responded only to changes in music feedback, we expected unidirectional causal relationships with a directional flow from feedback to the respective brain activity features $s\to \vec{f}$. Alternatively, brain activity modulations contributing to changes in music feedback should be reflected in causal relationships with a directional flow from respective brain activity features to the feedback $\vec{f}\to s$. Bi-directional causal relationships are of particular interest as they suggest features that interacted reciprocally with the music feedback, i.e., they both caused and responded to feedback modulations ($s\to \vec{f}$ & $\vec{f}\to s$). For these analyses, we first concatenated the observations from the action periods (60 observations per trial) for participants P01, P04, P05 during task → *happy*. We then computed the causal relationships between each feature and the feedback score resulting in 71x71 causal relationships. Causal relationships were computed using the Granger causal connectivity toolbox [60] with a maximal number of lags *nlags = 10* (corresponding to 5 sec) and a statistical Bonferroni-corrected threshold of *p* < 0.01. Fig 8 shows the results of our analysis. As a main result, bi-directional causal flow was discovered, particularly in gamma-activity over FC6. This provides support that the gamma-power related brain activity modulations observed in the previous analysis on brain activity modulations (see Fig 7) were (1) causing the feedback to change, and (2) caused by changes in the feedback, i.e. reciprocally interacted with the music feedback. Moreover, causal flow $s\to \vec{f}$ (reaction) spread from right temporal to anterior-central and posterior-central areas, whereas the causal flow $\vec{f}\to s$ (modulation) was mainly focused on FC6. Interestingly, no causal relationships were apparent for the significant modulations in frontal beta that were observed as a result of our previous analysis (see Fig 7). This suggests that the frontal beta activity did not cause the musical feedback to change, and was also not influenced by it. This suggests that the frontal beta activity modulations were rather a side effect, not directly contributing to the interaction, and may have been the result of participants concentrating on the task.

## 4 Discussion

### 4.1 Listening study

The listening study was conducted to investigate whether participants’ affective responses to generated musical patterns were in accordance with the corresponding valence and arousal settings of the automatic music generation system. The results confirmed a good match as indicated by exclusively positive within-participant correlation coefficients between self-assessed perceptual ratings of emotional affect and valence/arousal settings of the music generation algorithm. Despite the overall match between perceptual ratings and music generation settings, we observed high variance of ratings across subjects. The variance discovered between participants was probably due in large part to the variety of demographic backgrounds of the listeners (e.g., cultural background, music listening habits, musical training, etc.). Overall, the findings validated the functionality of the automatic music generation system as a parameterizable emotion stimulus and justified its utilization in the proposed closed-loop BCI architecture.

Closer inspection of the results revealed that arousal ratings match in general better than the valence ratings, as shown in Fig 5A and 5B. This is reflected in higher median across subject correlations and lower variations among subjects for the arousal ratings than for the valence ratings. This finding is in line with previous research; as stated by Yang et al. [61], across studies, researchers often find it is easier to predict or model arousal than valence. In their own study, Yang and colleagues reported best performance (as evaluated with R² statistics) for modeling arousal at 58.3%, but only 28.1% for valence, using a SVM regression model. Furthermore, one may observe in the current Listening study that arousal ratings were largely independent of valence music generation settings; but in contrast, valence ratings were somewhat dependent on the arousal music generation settings (Fig 5A). This dependency has also been found in other research (e.g., [62, 63]). As summarized in [64], Yoo and Lee (2011) found that arousal and valence were not completely independent from one another, even though they were two dimensions in the 2D Thayer-Russell space [63].

With regard to our automatic music generation system itself, we decided to implement harmonic mode in a valence-related order suggested by Schmuckler in 1989 [45] with lydian and ionian mode ranking first and second, respectively, in terms of positive valence. The results, however, indicated that ionian mode was rated with higher valence on average than lydian mode, suggesting that swapping these two modes in a future version of the automatic music generation system could result in a better match with perceptual valence ratings. Similarly, the influence of valence on arousal might be minimized in future applications of this work by modifying the functional relationships between the valence/arousal parameters and the music structural parameters. Both modifications can be implemented in a straightforward manner based on the perceptual ratings collected in this study. Finally, although participants were specifically asked to rate their experienced (induced) emotion, this task can be rather difficult for participants [17, 18, 65]. Therefore, we cannot exclude the possibility that the ratings of emotional affect were to some extent intertwined with perceived emotional expressiveness. This would make an interesting avenue for future research.

### 4.2 BCI study

The results of the BCI pilot study were a preliminary proof-of-concept that feedback modulations are possible using our proposed music-based BCI system. According to the participants’ reports, music feedback modulations were mainly performed by self-inducing the target emotions by recalling emotional memories. The analysis of resting versus action means of the score observations revealed that in all but two out of 10 participant-session combinations, the intended modulations were achieved. One of 5 participants achieved significant modulations in both tasks, whereas two other participants achieved significant modulations in one of the two given tasks. The analysis of relationships between modulation performance and mood rating prior to the experiment provided useful insight about why some of the participants performed well whereas others exhibited rather low performance. In particular, self-reports of feeling concentrated rather than distracted prior to the experiment significantly correlated with the feedback modulation performance during the task → sad. In general, high inter- and intra-participant variations were observed. This was reflected in the bias some participants developed towards one of the classes during the online application, which resulted in biased output by the BCI classification model. This phenomenon (classification bias) is common in the online application of BCIs and typically a result of shifts of feature distributions, e.g. due to electrodes drying, drowsiness, and attention variations.

Our investigation of brain activity modulations during one specific task showed significant differences for the participants who performed well in the experiment. Right hemisphere gamma power and frontal beta power modulations were found to be involved in the interaction task. While power modulations were most pronounced in frontal beta and right frontal gamma, Granger causality analysis suggested that only right frontal gamma contributed to the feedback modulations. Although a reduction of beta band activity was found during self-induced imagination of emotions, our causality analysis suggested that the frontal beta modulations did not significantly contribute to the interaction between brain states and emotion modulations. While previous authors have discovered positive correlations between beta band power over right temporal sites and valence [56], beta band activity could have been a side effect due to the participants concentrating on the task [66]. Regarding the gamma power modulations, the possibility that muscular activity (e.g. facial or neck muscle tension) contributed to the observed findings cannot be completely excluded. Nevertheless, gamma power modulations have been consistently reported in the literature in relation to emotion processing, based on a variety of experimental tasks and stimuli [57–59]. Onton and Makeig observed positive correlations between gamma-modulations over anterior temporal sites and emotional valence, which was also confirmed by Koelstra et al. [67]. Further, out of a set of features tested, Lin et al. [68] found temporal gamma-power to have the most discriminative power for the classification of emotional affect in music listening.

The literature therefore lends support to our hypothesis that subjects intentionally modulated the music feedback based on self-inducing corresponding emotions. Although these findings are promising, it is important to note that they are based on a relatively low number of participants. This, in conjunction with high inter- and intra-participant variations, prevents us from drawing strong conclusions from the pilot study. Nevertheless, the findings provide important insight into how the system works, and point towards future research directions. These considerations are addressed in the following section.

### 4.3 Future work

A follow-up study would be helpful to confirm the initial findings of the pilot study. Future work would most likely benefit from: (1) increasing the sample size, (2) collecting more data during the calibration phase (this would allow for more robust modeling and evaluation of the emotion classifier, and may counteract the observed classification bias), and (3) including automatic balancing of the task distribution during online application, or restricting the study to a single task (e.g., → happy) only. In addition, although the Emotiv EPOC system has been validated in numerous BCI studies, as discussed above, one could use a high-density research-grade EEG system for more robust localization of neural activity.

The preliminary results of the BCI study show that feedback modulations are possible via this music-based BCI system, and the brain activity analyses (especially the causality analysis) demonstrate how brain processes involved in the interaction can be isolated effectively. These findings are a proof-of-concept for our system, and more generally, provide a promising new approach to studying emotion regulation and related brain processes. Because our BCI system features continuity of interaction with an adaptive, emotionally evocative stimulus, it may be used to investigate the interaction dynamics between a listener and affective stimuli, and to model the corresponding brain processes. In this way, the emotion regulation system could be described and modelled as a ‘controller’ pursuing a particular ‘desired emotional state‘. Moreover, we believe in the applicability of our concept in neuro-rehabilitation of affective disorders, which we seek to explore in the future. Closing the loop via a brain-computer interface allows users to monitor their own brain processes, which can trigger neuroplasticity, recovery, or even enhancement of brain function.

Although our work did not evaluate the efficacy of the proposed BCI for use in neuro-rehabilitation, we believe that this would be a worthwhile direction, and that our concept is particularly advantageous over conventional approaches to this topic: The use of a single parametrizeable emotion stimulus allows flexible and convenient use in both BCI calibration and online application. As such, users are exposed with the same stimulus in both phases, allowing for tight calibration-to-application immediacy. In other words, the proposed approach allows for continuous interaction with an emotionally evocative stimulus (online application) based on the brain responses that were engaged during passive listening of the very same stimulus (calibration phase). To the best of the authors’ knowledge, no such integrated approach has been presented before in the domain of affective BCIs. This opens possibilities for the development of novel neuro-rehabilitation treatment protocols.

### 4.4 Concluding remarks

We have presented the concept, technical realization and evaluation of a novel non-invasive BCI to establish affective closed-loop brain interaction by means of a music-based emotion feedback. The BCI is based on an automatic music generation system to generate patterns of synthesized affective music employable as a continuously controllable emotion stimulus. In our first study, we successfully evaluated the affective quality of synthesized musical patterns generated with the automatic music generation system. A second feasibility study was conducted to examine affective closed-loop interactions in several participants. Preliminary results suggested that participants were able to intentionally modulate the musical feedback by self-inducing emotions (e.g., by recalling memories), an important finding for future studies in this line of research. We believe that this BCI system, including the music-generation system and technical framework, can be used as an effective new tool for investigating affective responses, emotion regulation, and the rehabilitation of affective disorders.
